# Cdc37 suppression induces plasma cell immaturation and bortezomib resistance in multiple myeloma via Xbp1s

**DOI:** 10.1038/s41389-020-0216-1

**Published:** 2020-03-05

**Authors:** Meirong Zang, Jiaojiao Guo, Lanting Liu, Fengyan Jin, Xiangling Feng, Gang An, Xiaoqi Qin, Yangbowen Wu, Qian Lei, Bin Meng, Yinghong Zhu, Yongjun Guan, Shuhui Deng, Mu Hao, Yan Xu, Dehui Zou, Minghua Wu, Lugui Qiu, Wen Zhou

**Affiliations:** 1 State Key Laboratory of Experimental Hematology, National Clinical Research Center for Blood Diseases, Institute of Hematology & Blood Diseases Hospital, Chinese Academy of Medical Science & Peking Union Medical College, Tianjin, China; 2 Department of Hematology, The Third Affiliated Hospital of Hebei Medical University, Shijiazhuang, China; 3 Cancer Research Institute, School of Basic Medical Science Key Laboratory of Carcinogenesis and Cancer Invasion, Ministry of Education; Key Laboratory of Carcinogenesis, National Health and Family Planning Commission, Central South University, Hunan, China; 4 Cancer Center, The First Hospital of Jilin University, Changchun, China; 5 Xiang Ya School of Public Health, Central South University, Changsha, Hunan China

**Keywords:** Oncogenes, Haematological cancer

## Abstract

Multiple myeloma (MM) is the second most prevalent hematologic malignancy. Although the use of bortezomib (BTZ) significantly improves MM therapy, intrinsic and acquired drug resistance to BTZ remains a major clinical problem. In this study, we find that Cdc37, a key co-chaperone of Hsp90, is downregulated in relapsed MM patients, especially after BTZ treatment, suggesting a link between Cdc37 and BTZ resistance. Suppression of Cdc37 or inhibition of Cdc37/Hsp90 association induces plasma cell dedifferentiation, quiescence of MM cells, and BTZ resistance in MM. Furthermore, we discover that Cdc37 expression correlates positively with Xbp1s, a critical transcription factor for plasma cell differentiation in MM samples. Depletion/inhibition of Cdc37 downregulates Xbp1s, while overexpression of Xbp1s in MM cell lines partially rescues plasma immaturation and BTZ resistance. It is suggested that Xbp1s may act as a key downstream effector of Cdc37. Experiments with a mouse model also demonstrate that Cdc37 inhibition promotes plasma cell immaturation, confers BTZ resistance, and increases MM progression in vivo. Together, we identify a critical factor and a new signaling mechanism that regulate plasma cell immaturation and BTZ resistance in MM cells. Our findings may constitute a novel strategy that overcomes BTZ resistance in MM therapy.

## Introduction

Multiple myeloma (MM) is a plasma cell neoplasia characterized by clonal expansion of malignant plasma cells and production of large amounts of monoclonal immunoglobulins (Ig)^1^. Despite the advances in the regimens of MM treatment, MM remains incurable due to intrinsic or acquired drug resistance^2^. Bortezomib (BTZ), a first-in-class selective proteasome inhibitor, has resulted in a significant improvement of MM treatment^3^. However, nearly all MM patients eventually relapse, and approximately one-third of MM patients are resistant to BTZ^2^. Clearly, a better understanding of the mechanism underlying BTZ resistance depends upon studies using the clinical samples, especially those with BTZ resistance.

We previously identified several highly expressed chromosome instability genes involved in drug resistance in sequential MM samples^4^. In addition, we also discovered Cdc37 as a gene that is downregulated with disease progression. Cdc37 is a key co-chaperone of Hsp90, acting as an adaptor to load protein kinases to the Hsp90 complex, to regulate the function of Hsp90 with temporal specificity and substrate selectivity, and to guide the stabilization and activation of protein kinases^5–7^. It has been shown that Cdc37 is required for chromosome segregation, cytokinesis, and proliferation in many normal cell types^8,9^. There is also a growing interest in the role of Cdc37 in malignancy. Previous studies have already demonstrated that Cdc37 is overexpressed in many types of solid cancers, including carcinoma of colon^10^, breast cancer^11^, and hepatocellular carcinoma^12^. Similarly, Cdc37 is significantly upregulated in MM cells with cyclinD1 overexpression^13^. Thus, inhibition of Cdc37 seems to be a promising approach to the treatment of cancer due to its multi-targeting nature. Indeed, inhibition of Cdc37 induces growth arrest in prostate cancer^14^. Consistently, destructing the trinity of CK2, Cdc37, and Hsp90 has been confirmed to induce MM cell apoptosis^15^. However, the precise role of Cdc37 in malignant transformation of plasma cells to MM remains unclear. Also, the pattern of Cdc37 expression in MM patients and the potential effect of pharmacological disruption of Cdc37–Hsp90 interaction remain undetermined. Such gaps in knowledge have limited the application of Cdc37 as a potential target for MM therapy.

In this study, we explored the role of Cdc37 in mediating MM progression and BTZ resistance in patient samples. We also investigated how Cdc37 might induce BTZ resistance in vitro and in vivo. Our study suggests that Cdc37 may serve as a reliable, clinically useful molecule that may be used for predicting drug resistance and developing novel therapeutic strategies in MM treatment.

## Materials and methods

### Patient samples

Human bone marrow samples were obtained from 60 newly diagnosed and 25 relapsed MM patients in the Department of Lymphoma and Myeloma, Institute of Hematology & Blood Disease Hospital, Chinese Academy of Medical Sciences, and Peking Union Medical College (Tianjin, China). Written informed consent was obtained from all participants. All studies with human samples were approved by the Medical Ethics Committee of Chinese Academy of Medical Sciences. Primary MM cells were isolated from the mononuclear cells of BM samples using CD138 microbeads (Miltenyi Biotec, Auburn, CA, USA).

### Cell culture

Human MM cell lines ANBL6 BTZ-sensitive (ANBL6.WT) and -resistant (ANBL6.BR) cells were kindly provided by Dr. Robert Orlowski (MD Anderson Cancer Center, Houston, TX, USA). RPMI-8226 Doxorubicin-resistant (RPMI-8226.Dox40) and -sensitive (RPMI-8226.WT) cells were provided by Dr. Yingdai Gao (State Key Laboratory of Experimental Hematology, Tianjin, China). The other MM cell lines, including MM.1S, MM.1R, KMS11, NCI-H929, RPMI-8226, U266, and ARP1, are previously reserved in the State Key Laboratory of Experimental Hematology (Tianjin, China) and Cancer Research Institute, Central South University (Hunan, China). All the cell lines were maintained in RPMI-1640 medium (Gibco, Thermo, USA), supplemented with 10% fetal bovine serum, 100 U/ml penicillin, and 100 μg/ml streptomycin. IL-6 (1 ng/ml) was added to the culture media for ANBL6.WT and ANBL6.BR, which were IL-6 dependent as previously described^16,17^. NCI-H929 and KMS11 were authenticated by STR profiling. NCI-H929, KMS11, and ARP1 were pathogens with negative detection of mycoplasma screened by PCR.

### Reagents

Cdc37 antibody (ab2800, ab109419) and CD49e (ab150361) were purchased from Abcam (MA, USA). Xbp1s antibody (#647501) was purchased from Biolegend (San Diego, CA, USA), and antibodies for Caspase-3 (#9662), Hsp90 (#5087), Nanog (#4903), p21(#2947), CyclinD1 (#2978), β-actin (#3700), GAPDH (#5174), and HRP-conjugated secondary antibodies (#7074, #7076) were purchased from Cell Signaling Technology (Danvers, MA, USA). Celastrol (C0869) was purchased from Sigma-Aldrich (St. Louis, MO, USA).

### Lentiviral production and transduction

Lentiviral shRNA clones targeting Cdc37 were purchased from Sigma-Aldrich. The designated plasmids were tested: TRCN0000116632 (5′-CCGGGCCCATTCAAGTCTCTGCTTTCTCGAGAAAGCAGAGACTTGAATGGGCTTTTTG-3′), TRCN0000116633 (5′-CCGGCGGCAGTTCTTCACTAAGATTCTCGAGAATCTTAGTGAAGAACTGCCGTTTTTG-3′), TRCN0000116634 (5′-CCGGCCGGCAGTTCTTCACTAAGATCTCGAGATCTTAGTGAAGAACTGCCGGTTTTTG-3′), TRCN0000116635 (5′-CCGGCCAGACAATCGTCATGCAATTCTCGAGAATTGCATGACGATTGTCTGGTTTTTG-3′), and TRCN0000116636 (5′-CCGGGACAGCCAATTACCTGGTCATCTCGAGATGACCAGGTAATTGGCTGTCTTTTTG-3′). TRCN0000116633 consistently functioned better than the other clones, and was referred as “Cdc37shRNA”. Packaging and envelope plasmids were purchased from Addgene (Cambridge, MA, USA). Human Cdc37 and Xbp1s ORF lentiviral expression clone and virus package system was purchased from GeneCopoeia (Rockville, MD, USA). Recombinant lentivirus was produced in 293T cells. For lentiviral transduction, lentiviral-containing media and polybrene (8 μg/ml) were added into culture media containing nonattached cells. After 12 h media was replaced with fresh media. Cells expressing Cdc37shRNA were selected with puromycin (2.5 μg/ml), while Cdc37- and Xbp1s-overexpression cells were selected with flow cytometry 5 days later.

### Flow cytometry analysis

For cell cycle analysis, harvested cells were fixed in cold 70% ethyl alcohol for 12 h at 4 °C, then resuspended with PBS, and stained with propidium iodide (PI) (BD Biosciences, NJ, USA) for 30 min at 37 °C. DNA content was determined by flow cytometry. For apoptosis analysis, AnnexinV/7AAD (BD Biosciences) or AnnexinV/PI Kit (BD Biosciences) was used to label apoptotic cells according to the manufacturer’s instructions. The results were analyzed using FlowJo software. These experiments were conducted independently at least three times.

Bone marrow samples from MM patients were obtained at the time of routine clinical procurement following informed consent. Cells were collected in heparinized tubes and processed by Ficoll gradient. Tumor cells were enriched on BD Biosciences FACS AriaII by FSC–SSC characteristics, negative selection for CD14 (BD Biosciences, #561383) and CD2 (BD Biosciences, #555328), and then sorted into subpopulations on the basis of CD38 (BD Biosciences, #555459) and CD138 (BD Biosciences, #561704) status for CD38^−^CD138^−^ pre-plasmablasts, CD38^+^CD138^−^ plasmablasts, and CD38^+^CD138^+^plasma cells^18^.

### Quantitative real-time PCR (qRT-PCR)

Total RNA was isolated using Trizol reagent (Invitrogen, Carlsbad, CA, USA) and was reverse-transcribed using M-MLV reverse transcriptase cDNA synthesis Kit (Invitrogen) according to the manufacturer’s instructions. The cDNA and specific primers were mixed with SYBR Premix Ex Taq^TM^ (2×) (Takara, China); then qRT-PCR was performed using ABI Prism 7500. The expression of the target gene relative to GAPDH was calculated using the 2^–ΔΔCT^ method. These experiments were repeated independently at least three times. The specific primers were listed as follows: Cdc37 (5′-TGAAGACGAGACGCACC-3′ and 5′-TCAGTTTCCTCTGGCACTCG-3′), Xbp1s (5′-GAGTCCGCAGCAGGTG-3′ and 5′-CTCTGGGGAAGGGCATTTGA-3′), Oct4 (5′-GCCCGAAAGAGAAAGCGAACC-3′ and 5′-CCCCCTGAGAAAGGAGACCCA-3′), Nanog (5′-CCGAAGAATAGCAATGGTGTGAC-3′ and 5′-GGACTGGATGTTCTGGGTCTGGT-3′), and GAPDH (5′-GAAGGTGAAGGTCGGAGTC-3′ and 5′-GAAGATGGTGATGGGATTTC-3′).

### Western blot analysis

Western blot was performed on whole-cell lysates extracted from cells. Briefly, protein samples were separated using sodium dodecyl sulfate–polyacrylamide gel electrophoresis and transferred into polyvinylidene fluoride membranes. The membranes were incubated overnight at 4 °C with primary antibody. The respective horse-radish peroxidase (HRP)-conjugated secondary antibodies were added, and protein signals were developed with enhanced chemiluminescence regents (Thermo Scientific, MA, USA). The images were obtained and analyzed using ChemiDoc^TM^ XRS + System (Bio-Rad, Hercules, CA, USA). Western blot analysis was repeated independently at least twice.

### Immunocytochemistry (ICC) analysis

Purified primary MM cells were spun down on glass slides and then evaluated for Cdc37 protein expression by ICC. A semiquantitative scoring system was applied. One hundred of MM cells were assessed for every slide. The Cdc37 staining was graded according to the intensity of staining (0 negative, 1 weak, 2 moderate, and 3 strong) and the percentage of positive cells. The final score for every case was calculated as 1 × (number of weak-staining cells)% + 2 × (number of moderate-staining cells)% + 3 × (number of strong-staining cells)%.

### Immunofluorescence analysis

Sorted cells were spun down on glass slides, and then fixed with −20 °C acetone/methanol (volume 1:1) for 10 min. Then, cells were blocked by immersion in 5% goat serum for 60 min. Cells were incubated with the primary antibodies overnight at 4 °C, followed by incubation with secondary antibodies for 30 min at room temperature in the dark. Nuclei were stained with DAPI. The slides were sealed with nail polish. Fluorescence images were acquired by fluorescence microscope.

### ELISA analysis

Human Kappa ELISA Quantitation Set (Bethyl Laboratories, AL, USA), Mouse IgG2b ELISA Quantitation Set (Bethyl Laboratories, AL, USA), and ELISA Starter Accessory Kit (Bethyl Laboratories, AL, USA) were used to determine Ig secretion according to the manufacturer’s instructions. The results were measured based on the absorbance (450 nm) with a microtiter plate reader, and the content was determined using CurveExpert 1.4 software.

### 5TMM mouse model study

In this study, 24 male C57BL/KaLwRij mice of 10–12 weeks old were purchased from Harlan Laboratories Inc. (Indianapolis, IN, USA). Mice were housed following the conditions approved by the Ethical Committee for Animal Experiments of Institute of Hematology & Blood Diseases Hospital. To prepare MM mice models, 5T33mmvt cells (3 × 10^6^ cells in 300 µl of PBS) were injected into C57BL/KaLwRij mice through the tail vein. After 1 week, mice were randomly divided into four groups (six mice in each group), and then treated twice a week with celastrol (0.5 mg/kg), BTZ (1 mg/kg), celastrol plus BTZ (0.5 mg/kg plus 1 mg/kg), and PBS, respectively.

After 2 months, the mice were euthanized, while femurs, tibias, and iliac crest were harvested immediately and placed in cold ffPBE buffer (PBS + 2% FBS + 2 mM EDTA). Bone marrow cells were obtained through rinsing femurs, tibias, and iliac crest with fresh and sterile PBS. The following antibodies were used to stain the cells: CD138-PE (clone 281-2, Biolegend), B220/CD45RA-Percp-Cy5.5 (clone RA3-6B2, Biolegend), CD3-FITC (clone 17A2, Biolegend), and IgM-PE-Cy7 (eB121-15F9, Biolegend). Flow cytometry analyses were performed on FACS Canto II. Data were analyzed with FlowJo Software.

Extramedullary myeloma tissues removed from mice were subjected to immunofluorescence staining for CD138 (ab128936, Abcam) and Xbp1s (sc-7160, Santa Cruz). Microscopic images were captured with UltraVIEW Vox confocal microscopy.

### Accession numbers

Microarray data sets were deposited in the National Center for Biotechnology Information’s Gene Expression Omnibus (GEO) database (http://www.ncbi.nlm.nih.gov/geo) with the accession number GSE2658 and GSE19554.

### Statistical analysis

Values represented were mean ± standard deviation (SD) from at least three separate experiments. Statistical significance of the data was determined using the Student’s unpaired *t* test. *p* < 0.05 was considered statistically significant. Homogeneity of variance test has been performed (*p* > 0.05) prior to the *t* test. Statistical analysis was performed using the IBM SPSS 19.0 software.

## Results

### Decreased Cdc37 expression is linked to BTZ resistance in MM

**Fig. 1:**
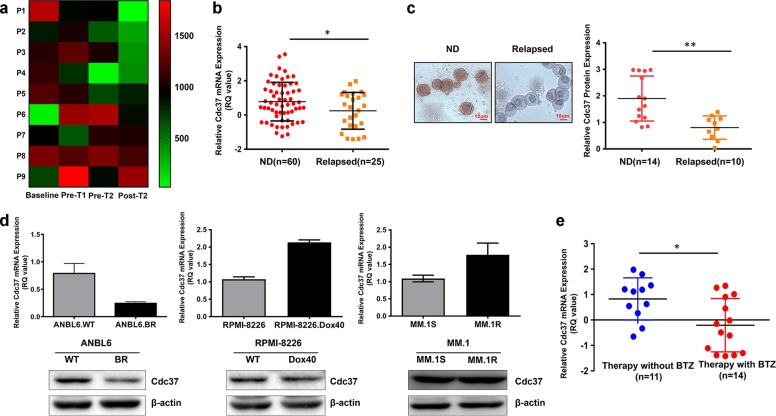
Low expression of Cdc37 is linked to BTZ resistance in multiple myeloma. **a** Heatmap showing Cdc37 mRNA expression in a sequential MM sample set [at diagnosis (Baseline), pre first transplantation (Pre T1), pre second transplantation (Pre T2), and post second transplantation (Post T2)], data obtained from GSE19554. **b** Cdc37 mRNA expression was detected in CD138^+^ cells from 60 newly diagnosed MM patients (ND) and 25 relapsed cases (Relapsed). The relative Cdc37 mRNA expression in ND and relapsed MM patients was 0.78 ± 1.13 and 0.25 ± 1.07, respectively (*p < 0.05). **c** Cdc37 protein expression in primary MM cells was examined with ICC. Left panel: MM cells derived from 14 newly diagnosed patients (ND) and 10 relapsed patients (Relapsed) were subjected to ICC for Cdc37 protein expression, and representative cases are shown. Right panel: The semiquantitative scoring is shown. The relative Cdc37 protein expression in ND and relapsed MM patients was 1.90 ± 0.85 and 0.81 ± 0.44, respectively (**p < 0.01). **d** Cdc37 mRNA expression (upper) and protein level (lower) were examined in ANBL6.WT/ANBL6.BR cells (left), RPMI-8226/RPMI-8226.Dox40 cells (middle), and MM.1S/MM.1R cells (right) by qRT-PCR and western blot. **e** Cdc37 mRNA expression was detected in CD138^+^ cells from 14 patients relapsed after BTZ-based treatment, while 11 cases relapsed after other therapy without BTZ. The relative Cdc37 mRNA expression was −0.21 ± 1.05 and 0.83 ± 0.83, respectively (*p < 0.05).

To assess potential association between Cdc37 expression and response to BTZ treatment, we analyzed previously published gene expression profile data of nine sequential MM samples (GSE19554)^4^. The gene expression analysis revealed that five MM patients (P1–P5) underwent significant downregulation of Cdc37 at one or more time points after treatments compared with the baseline (Fig. 1a). In addition, we examined Cdc37 gene expression in plasma cells derived from 60 newly diagnosed (ND) and 25 relapsed MM patients using qRT-PCR. As shown in Fig. 1b, Cdc37 was highly expressed in newly diagnosed MM patients compared with relapsed counterparts. Consistently, Cdc37 protein was also downregulated in relapsed MM patients (Fig. 1c), suggesting that the reduced Cdc37 level is related to the response of MM patients to treatment(s). To further explore which clinical treatment(s) confers the decreased Cdc37 expression, we examined Cdc37 expression in three drug-resistant MM cell lines, including BTZ-resistant cell line (ANBL6.BR), doxorubicin-resistant cell line (RPMI-8226.Dox40), and dexamethasone- resistant cell line (MM.1R). Interestingly, only BTZ-resistant cell line ANBL6.BR had a low Cdc37 expression compared with its counterparts (Fig. 1d). To further determine whether a low Cdc37 level is linked to BTZ resistance in MM samples after clinical treatment, we examined Cdc37 expression in 25 relapsed MM patients, including 14 BTZ-treated patients and 11 patients with other treatment. The therapeutic regimen and clinical characteristics of these patients are listed in Table 1.

Table 1 indeed, relapsed patients after BTZ treatment displayed much lower Cdc37 expression compared with relapsed patients after other treatments (Fig. 1e). Taken together, these results revealed a close link between Cdc37 expression and disease states, especially in patients exposed to BTZ-based therapy.

**Table 1:** The clinical characteristics of MM patients with different treatments.

Patients’ characteristics	Relapse after BTZ-based therapy (*N* = 14)	Relapse after therapy without BTZ (*N* = 11)	*p* Value
Male (*n*/*N*)	7/14	7/11	0.495
Median of age (y)	61	58	0.494
Albumin (g/L)	34.51 ± 6.61	33.30 ± 6.89	0.716
β2-microglobulin (mg/L)	8.83 ± 7.80	6.26 ± 2.57	0.486
LDH (U/L)	259.29 ± 195.21	216.67 ± 80.79	0.616
Hemoglobin (g/L)	83.86 ± 13.00	84.33 ± 18.06	0.947
Creatimine (μmol/L)	110.11 ± 72.68	92.38 ± 41.93	0.587
Ca^2+^ (mmol/L)	2.21 ± 0.15	2.20 ± 0.20	0.959
Subtype of MM (n/N)			0.853
IgG	3/14	4/10	
IgA	5/14	3/10	
IgD	1/14	1/10	
Light-chain type—kappa	2/14	1/10	
Light-chain type—lambda	3/14	1/10	
Karyotype (n/N)			0.501
Diploidy	5/12	2/3	
Hyperdiploidy	3/12	1/3	
Hypodiploidy	4/12	0/3	
FISH			
IgH translocation (n/N)	8/11	2/5	0.210
t (11;14)	2/6	–	
t (4;14)	1/6	–	
t (14;16)	1/5	–	
13q deletion	9/11	3/5	0.350
p53 deletion	5/12	0/5	0.086
Proplasmacyte >30% (bone marrow aspiration)	7/12	1/8	0.040
Treatment option with BTZ			
BD	2/14	–	
BCD	7/14	–	
PAD	1/14	–	
VMP	1/14	–	
VDD	3/14	–	
RD	–	6/11	
TAD	–	4/11	
DECP	–	1/11	

*LDH* lactate dehydrogenase, *FISH* fluorescence in situ hybridization, *BD* bortezomib and dexamethasone, *BCD* bortezomib, cyclophosphamide, and dexamethasone, *PAD* bortezomib, epirubicin, and dexamethasone, *VMP* bortezomib, melphalan, and dexamethasone, *VDD* bortezomib, liposome, doxorubicin, and dexamethasone, *RD* lenalidomide and dexamethasone, *TAD* thalidomide, epirubicin, and dexamethasone, *DECP* cisplatin, etoposide, cyclophosphamide, and dexamethasone.

### Suppression of Cdc37 induces BTZ resistance in MM cells

**Fig. 2:**
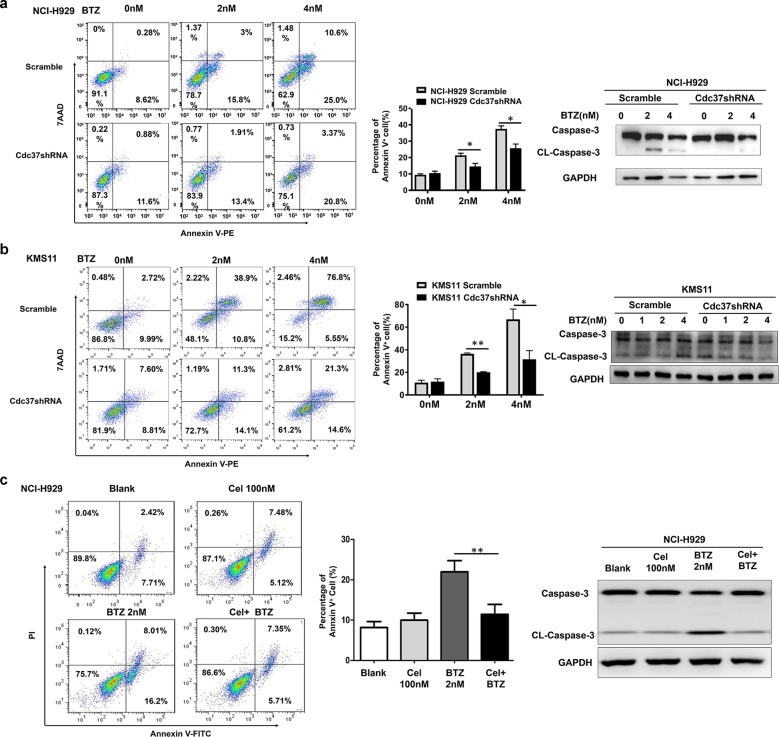
Suppression of Cdc37 induces BTZ resistance in MM cells. **a** NCI-H929 cells were infected with scramble (NCI-H929 Scramble) and Cdc37shRNA (NCI-H929 Cdc37shRNA) lentivirus. Left panel: The apoptosis rate was detected by flow cytometry. Middle panel: The statistical analysis of apoptosis cell distribution from three repeated experiments (*p < 0.05, **p < 0.01). Right panel: Caspase-3, CL-Caspase-3, and GAPDH were detected by western blot analysis. **b** KMS11 cells were infected with scramble (KMS11 Scramble) and Cdc37shRNA (KMS11 Cdc37shRNA) lentivirus. Left panel: The apoptosis rate was detected by flow cytometry. Middle panel: The statistical analysis of apoptosis cell distribution from three repeated experiments (*p < 0.05, **p < 0.01). Right panel: Caspase-3, CL-Caspase-3, and GAPDH were detected by western blot analysis. **c** NCI-H929 cells were treated with celastrol (Cel), BTZ, or their combination for 24 h. Left panel: The apoptosis analysis with flow cytometry. Middle panel: The statistical analysis of apoptosis cell distribution was performed from three repeated experiments (*p < 0.05, **p < 0.01). Right panel: Caspase-3, CL-Caspase-3, and GAPDH were detected by western blot analysis.

To explore whether the relationship between low expression of Cdc37 and BTZ resistance was functional, we depleted Cdc37 in MM cell lines NCI-H929 and KMS11 via shRNA-mediated knockdown (Supplemental Figs. 1A and 1B). We found that Cdc37 depletion caused a reduced apoptosis rate, accompanied with a decreased caspase-3 protein level after BTZ treatment (Fig. 2a, b). Previous studies demonstrated that celastrol (Cel), a natural triterpene compound isolated from the traditional Chinese medicinal plant *Tripterygium wilfordii*, disrupts the Hsp90–Cdc37 interaction^19–21^, leading us to confirm its Hsp90–Cdc37 inhibition effect in NCI-H929 MM cells (Supplemental Figs. 1C and 1D). Consistent with the effect of Cdc37 depletion, celastrol also reduced the apoptosis rate in NCI-H929 cells after BTZ treatment (Fig. 2c). Thus, Cdc37 suppression is a feature of BTZ resistance in MM cells.

### Cdc37 expression correlates with that of Xbp1s

**Fig. 3:**
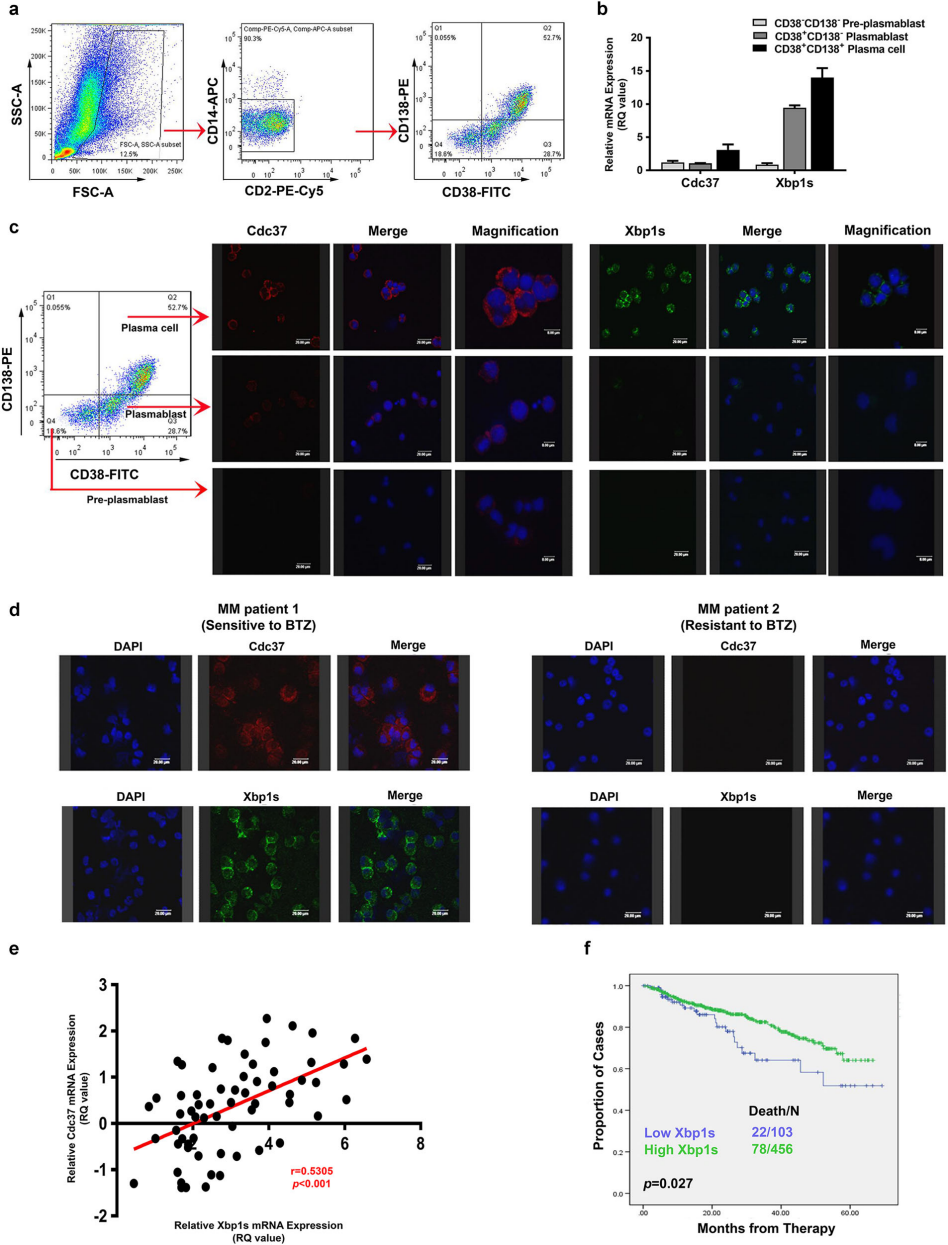
Cdc37 is gradually upregulated during plasma cell maturation and positively correlates with Xbp1s. **a** Bone marrow MM cells were enriched by sequential FACS of lymphoid FSC/SSC, negative selection for CD2 (T and NK cells) and CD14 (monocytes), as well as gated into a subpopulation by CD38 and CD138 status. **b** Sorted CD38^−^CD138^−^ pre-plasmablast, CD38^+^CD138^−^ plasmablast, and CD38^+^CD138^+^ plasma cells were subjected to qRT-PCR analysis for gene expression of Cdc37 and Xbp1s. **c** CD38^−^CD138^−^ pre-plasmablast, CD38^+^CD138^−^ plasmablast, and CD38^+^CD138^+^ plasma cells were subjected to immunofluorescence analysis for protein expression of Cdc37 and Xbp1s. **d** Immunofluorescence analysis of Cdc37 and Xbp1s in CD138^+^ cells derived from a newly diagnosed patient responding to BTZ-based treatment (MM patient 1) and a patient displaying resistance to BTZ (MM patient 2). **e** Correlation analysis of gene expression of Cdc37 and Xbp1s from 45 newly diagnosed and 18 relapsed MM patients from qRT-PCR data. **f** Data obtained from GSE2658 showing prognostic relevance of Xbp1s mRNA on MM. Kaplan–Meier analyses revealed that MM patients with low expression of Xbp1s had inferior event-free survival in newly diagnosed TT2 and TT3 cohorts.

Earlier studies with cDNA array showed that Cdc37 is upregulated in primary B cells in response to BCR signaling, implying that Cdc37 is involved in B-cell activation and differentiation^22^. It is also well established that downregulation of plasma cell maturation molecules, especially Xbp1s, results in decreased Ig synthesis and a lower stress level of proteasome load, which might render MM cells resistant to BTZ^18,23–25^. We therefore explored whether Cdc37 was associated with B-cell differentiation and plasma cell maturation. First, we isolated B-lineage cells and plasma cells from BM mononuclear cells based on CD38/CD138 expression as previously described^18^ from five newly diagnosed MM patients and one relapsed MM patient after five cycles of BTZ-based treatment. We then obtained three subpopulations, including CD38^−^CD138^−^ pre-plasmablasts, CD38^+^CD138^−^ plasmablasts, and CD38^+^CD138^+^plasma cells, representing different stages of plasma cell differentiation and maturation (Fig. 3a), and subsequently determined Cdc37 and Xbp1s expression in these three subpopulations by qRT-PCR and immunofluorescence. Both Xbp1s and Cdc37 were gradually upregulated during plasma cell maturation in 4/5 newly diagnosed patients (Fig. 3b, c). Notably, Cdc37 expression was barely detectable in CD38^+^CD138^+^ plasma cells in the relapsed patient (data not shown).

Having revealed a link between Cdc37 and Xbp1s during plasma cell differentiation/maturation, we asked whether they were also associated with BTZ resistance. First, we found a reduced expression of Xbp1s and Chop, the critical transcription factors mediating plasma cell differentiation, in BTZ-resistant cell line ANBL6.BR (Supplemental Fig. 2), suggesting that Cdc37 and Xbp1s may be involved in BTZ resistance through plasma cell differentiation. Then we examined their expression in BTZ-sensitive or -resistant MM patients. As shown in Fig. 3d, immunofluorescence analysis showed that primary MM cells from BTZ-sensitive patient (MM patient 1) expressed a high level of Cdc37 and Xbp1s, while cells from a BTZ-resistant patient (MM patient 2) exhibited a low level of Cdc37 and Xbp1s. We then examined Xbp1s and Cdc37 gene expression simultaneously in MM cells from 45 newly diagnosed and 18 relapsed patients by qRT-PCR, and discovered a positive correlation between Cdc37 and Xbp1s in CD138^+^ cells from MM patients (Fig. 3e). Moreover, gene expression profile data indicated that MM patients with low Xbp1s expression were associated with inferior clinical outcome (Fig. 3f)^26^. In brief, Cdc37 is closely linked to Xbp1s during plasma cell differentiation/maturation and MM development.

### Suppression of Cdc37 induces immaturity of plasma cells

**Fig. 4:**
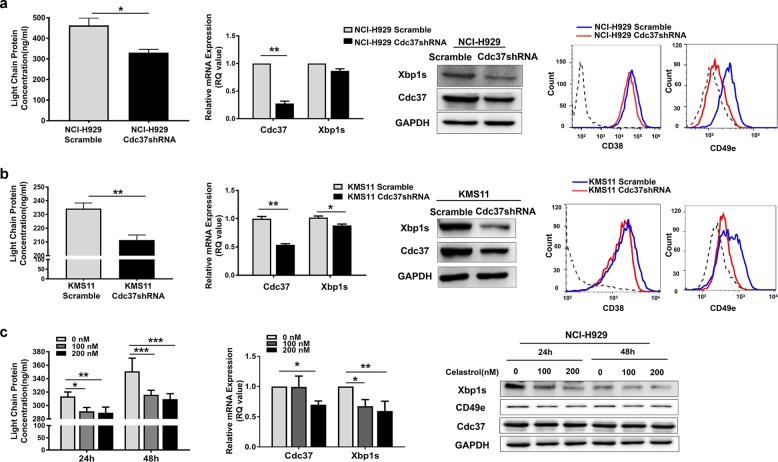
Suppression of Cdc37 induces immaturity of plasma cells. **a** First panel: The cell culture supernatant of NCI-H929 Scramble and Cdc37shRNA cells was subjected to ELISA analysis for the concentrations of human immunoglobulin light-chain proteins (*p < 0.05). Second panel: Total RNA of NCI-H929 Scramble and Cdc37shRNA cells was isolated and subjected to qRT-PCR analysis for Cdc37 and Xbp1s (**p < 0.01). Third panel: Whole-cell lysates of NCI-H929 Scramble and Cdc37shRNA cells were subjected to western blot analysis for Cdc37 and Xbp1s. Fourth panel: Flow cytometric analysis of surface CD38 and CD49e on NCI-H929 Scramble and Cdc37shRNA cells. **b** First panel: The cell culture supernatant of KMS11 Scramble and Cdc37shRNA cells was subjected to ELISA analysis for the concentrations of human immunoglobulin light-chain proteins (**p < 0.01). Second panel: Total RNA of KMS11 Scramble and Cdc37shRNA cells was isolated and subjected to qRT-PCR analysis for Cdc37 and Xbp1s (*p < 0.05, **p < 0.01). Third panel: Whole-cell lysates of KMS11 Scramble and Cdc37shRNA cells were subjected to western blot analysis for Cdc37 and Xbp1s. Fourth panel: Flow cytometric analysis of surface CD38 and CD49e on KMS11 Scramble and Cdc37shRNA cells. **c** NCI-H929 cells were treated with 100 and 200 nM celastrol for 24 and 48 h, respectively. Left panel: The cell culture supernatant was subjected to ELISA for the concentration of human Ig light-chain proteins (*p < 0.05, **p < 0.01, ***p < 0.001). Middle panel: The total RNA was isolated and subjected to qRT-PCR analysis for Cdc37 and Xbp1s (*p < 0.05, **p < 0.01). Right panel: The whole-cell lysates were subjected to western blot analysis for Xbp1s, CD49e, and Cdc37.

The close relationship between Cdc37 and Xbp1s led us to ask whether Cdc37 also functions in plasma cell differentiation and maturation. We found that knockdown of Cdc37 by shRNA resulted in decreased secretion of Ig light chain and repressed expression of plasma cell maturation markers, such as Xbp1s, CD38, and CD49e^27,28^ in NCI-H929 cells (Fig. 4a) and KMS11 cells (Fig. 4b). We then disrupted the Cdc37–Hsp90 complex in NCI-H929 cells by celastrol. As shown in Fig. 4c, similar results were obtained in this process. Moreover, celastrol also led to downregulation of plasma cell maturation markers in RPMI-8226 and U266 cells (Supplementary Fig. 3).

### Suppression of Cdc37 increases quiescent MM cell populations

**Fig. 5:**
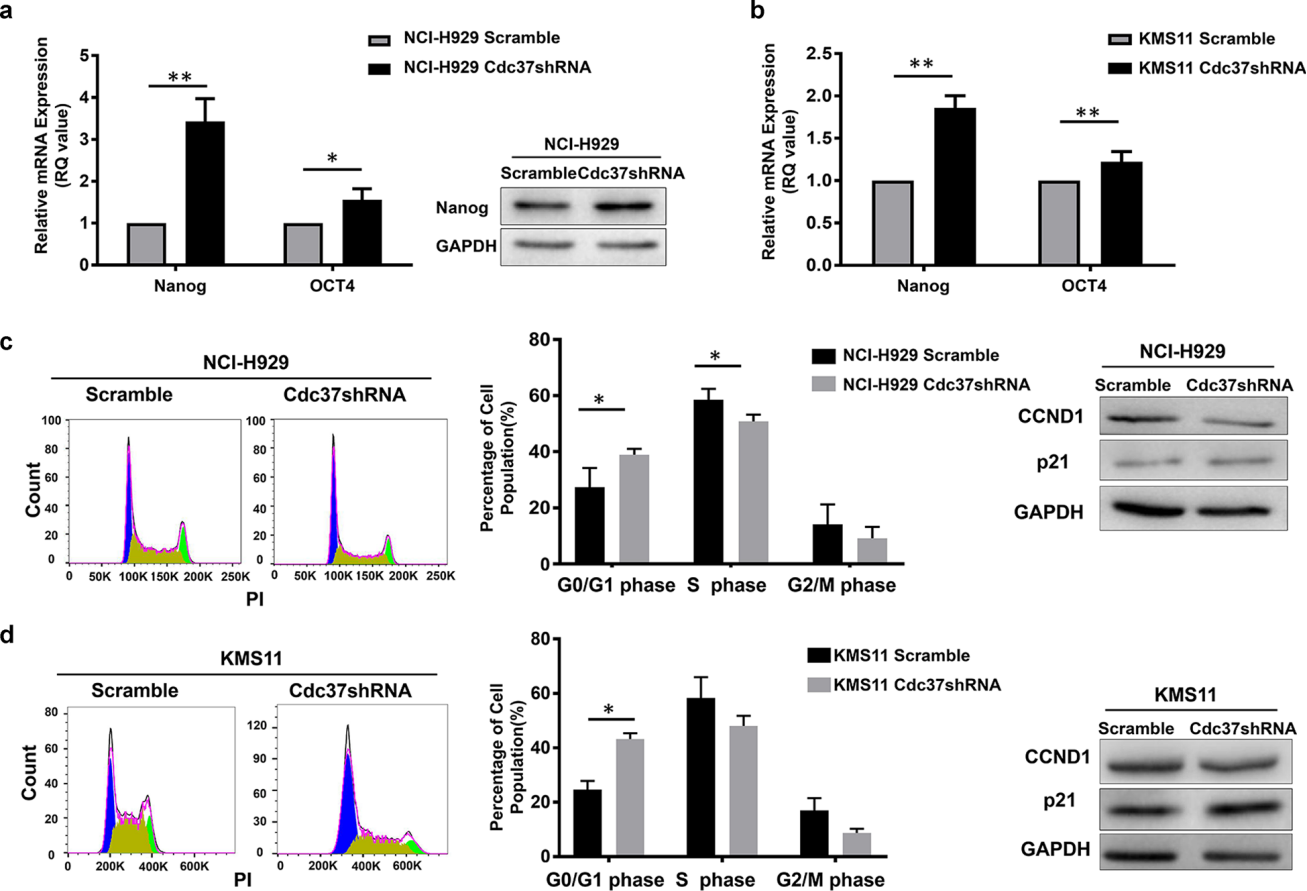
Suppression of Cdc37 increases the quiescent MM populations. **a** The mRNA and protein level of Nanog and Oct4 were detected in NCI-H929 Scramble and Cdc37shRNA cells by qRT-PCR and western blot (*p < 0.05, **p < 0.01). **b** The mRNA expression of Nanog and Oct4 was detected in KMS11 Scramble and Cdc37shRNA cells by qRT-PCR (**p < 0.01). **c** NCI-H929 Scramble and Cdc37shRNA cells were tested for the cell cycle. Left panel: The representative cell cycle analysis from flow cytometry. Middle panel: The statistical analysis of cell cycle distribution from three repeated experiments (*p < 0.05). Right panel: Whole-cell lysates were subjected to western blot for p21 and CyclinD1 (CCND1). **d** KMS11 Scramble and Cdc37shRNA cells were tested for the cell cycle. Left panel: The representative cell cycle analysis from flow cytometry. Middle panel: The statistical analysis of cell cycle distribution from three repeated experiments (*p < 0.05). Right panel: Whole-cell lysates were subjected to western blot for p21 and CyclinD1 (CCND1).

Several reports have shown that MM cells with an immature phenotype likely maintain a stem cell-like transcriptional program^29–32^. These stem cell-like cells are more quiescent and resistant intrinsically to therapy. We then assessed the effect of Cdc37 depletion on the “stemness” of the cells by examining the level of stem cell-specific transcription factors, including Nanog and Oct4. We observed significant upregulation of Nanog and Oct4 in Cdc37-depleted NCI-H929 cells (Fig. 5a) and KMS11 cells (Fig. 5b).

The stem cell-like MM cells that survive BTZ treatment usually enter quiescence, as characterized by p21 upregulation and cell cycle arrest^33^. We indeed found that Cdc37 depletion led to G0/G1-phase arrest, and at the protein level, Cdc37 depletion decreased cyclinD1, while it increased p21 in NCI-H929 (Fig. 5c) and KMS11 cells (Fig. 5d).

### Xbp1s acts as a downstream target of Cdc37

**Fig. 6:**
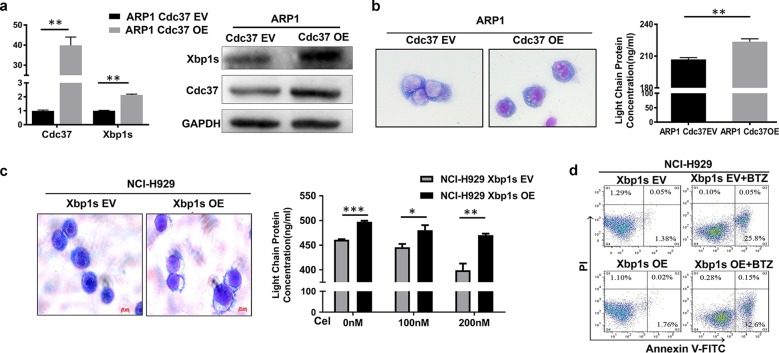
Overexpression of Xbp1s partially rescues BTZ resistance caused by Cdc37 suppression. **a** ARP1 cells were infected with empty vector (ARP1 Cdc37 EV) and Cdc37 ORF (ARP1 Cdc37 OE) lentivirus. The gene and protein level of Cdc37 and Xbp1s were detected in ARP1 Cdc37 EV and ARP1 Cdc37 OE cells by qRT-PCR and western blot. **b** Left panel: The cytomorphological changes of ARP1 Cdc37 EV and ARP1 Cdc37 OE cells. Right panel: The cell culture supernatant was subjected to ELISA analysis for the concentration of human immunoglobulin light-chain proteins (**p < 0.01). **c** NCI-H929 cells were infected with empty vector (NCI-H929 Xbp1s EV) and Xbp1s ORF (NCI-H929 Xbp1s OE) lentivirus. Left panel: The cytomorphological changes were performed in NCI-H929 Xbp1s EV and Xbp1s OE cells after the treatment with 200 nM celastrol for 72 h. Right panel: NCI-H929 Xbp1s EV and Xbp1s OE cells were treated with 100 and 200 nM celastrol, respectively for 48 h, and the concentration of human Ig light-chain proteins in the culture supernatant was determined by ELISA (*p < 0.05, **p < 0.01, ***p < 0.001). **d** NCI-H929 Xbp1s EV and Xbp1s OE cells were pretreated with 100 nM celastrol for 48 h, and then were treated with 2 nM BTZ for 24 h. The cells were subjected to apoptosis analysis.

We next explored the functional relationship between Cdc37 and Xbp1s. We showed earlier that Cdc37 depletion or inhibition led to downregulation of Xbp1s. Conversely, we do not know whether overexpression of Cdc37 could upregulate Xbp1s. First, we detected the expression of Cdc37 in ten MM cell lines, including NCI-H929, KMS11, RPMI-8226, U266, and ARP1. We found that Cdc37 was relatively low expressed in ARP1 cells (Supplemental Fig. 4). Then, we transfected Cdc37 ORF plasmid in ARP1 cells, and found that Xbp1s was upregulated after Cdc37 was overexpressed (Fig. 6a). We next confirmed that overexpression of Cdc37 led to MM cells to become more mature in morphology and to secrete more Ig light chains (Fig. 6b). It raised the possibility that Xbp1s may act as a downstream effector of Cdc37. We next hypothesized that overexpression of Xbp1s may rescue the effect of Cdc37 inhibition/depletion in MM cells. Consistent with this, overexpression of Xbp1s indeed converted cells with Cdc37 inhibition to mature plasma cells, as evidenced by morphological changes and an increase in Ig light-chain secretion (Fig. 6c). In addition, overexpression of Xbp1s partially restored BTZ-induced cytotoxicity in Cdc37-inhibited MM cells, as demonstrated by an increased rate of apoptosis (Fig. 6d). Together, these results suggest that Xbp1s may act as a key downstream target of Cdc37 in MM cells.

### Disruption of Cdc37 leads to BTZ resistance in vivo

**Fig. 7:**
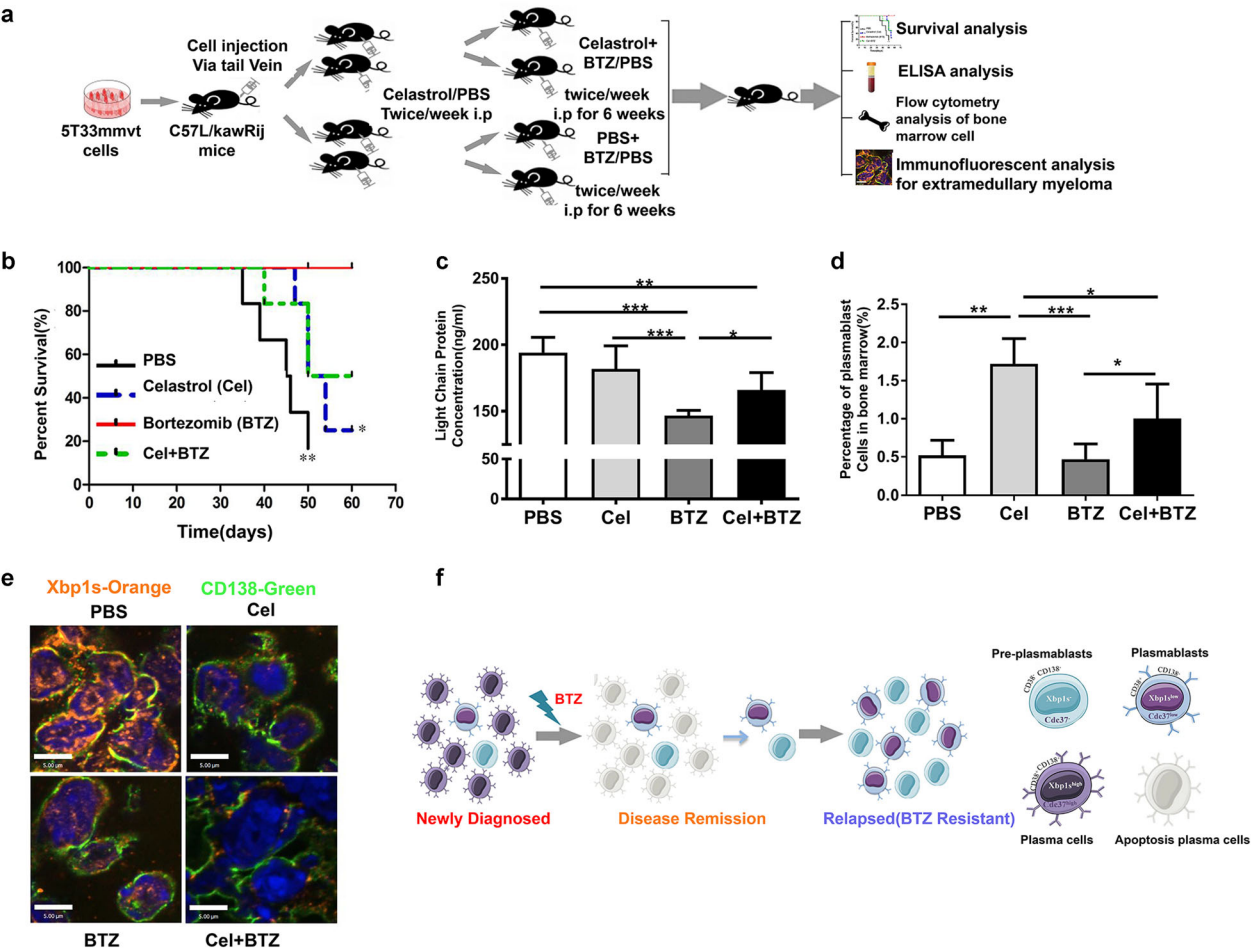
Disruption of Cdc37 leads to BTZ resistance in vivo. **a** The schematic diagram of the 5T33MMvt-KaLwRij model. **b** Kaplan–Meier analyses of overall survival of different experimental groups. **c** The concentrations of Ig light-chain proteins in the serum of different groups were determined with ELISA (*p < 0.05, **p < 0.01, ***p < 0.001). **d** B220^+^CD138^+^IgM^+^ plasmablast cells from mice bone marrow were selected with flow cytometry, and the percentages of plasmablast cells were calculated in the respective groups (*p < 0.05, **p < 0.01, ***p < 0.001). **e** Extramedullary myeloma tissues removed from 5T33MMvt-KaLwRij mice were subjected to immunostaining for CD138 (green) and Xbp1s (orange). **f** The model of our hypothesis in this work.

To confirm the role of Cdc37 in BTZ resistance in vivo, we employed the 5T33MMvt-KaLwRij mouse model. One week after injection of 5T33mmvt cells, mice were divided into four groups and administered with PBS, celastrol, BTZ, and celastrol plus BTZ, respectively (Fig. 7a). BTZ treatment significantly extended mouse survival (Fig. 7b) and delayed tumor progression, as evidenced by a decreased level of serum Ig light chain (Fig. 7c). However, the therapeutic effect of BTZ on MM was prevented by celastrol, thus confirming the role of Cdc37 in BTZ resistance. To determine the effect of Cdc37 inhibition on plasma cell immaturity in vivo, we analyzed the B220^+^CD138^+^IgM^+^ cells from the mouse BM using flow cytometry, because they are accepted as the BM plasmablasts^34–36^. Administration of celastrol increased the percentage of plasmablasts compared with the PBS, BTZ, and celastrol plus BTZ group. Notably, there was also a significant increase in the percentage of plasmablasts in the celastrol plus BTZ group compared with the BTZ group (Fig. 7d). Because 5T33MMvt cell line is a stroma-independent variant of 5T33MMvv cell line, nearly all mice developed extramedullary myeloma. We next removed the extramedullary tissues and analyzed them using CD138 and Xbp1s immunofluorescence. As shown in Fig. 7e, celastrol significantly decreased the level of Xbp1s and CD138 in vivo.

## Discussion

Since BTZ was introduced to MM therapy, it has significantly improved the survival of MM patients^37^. Despite the high initial response rate to BTZ, relapse remains inevitable in the majority of MM patients. Some relapsed patients maintain sensitivity to further BTZ-based therapy, while some develop refractory diseases due to “acquired” drug resistance. However, no reliable diagnostic predictors are available to determine whether a patient will respond to BTZ before the treatment starts. Therefore, novel strategies that can specifically target drug-resistant cells, as well as biomarkers that can predict BTZ sensitivity, are critically needed. Here we report the biological features of Cdc37 in BTZ-resistant MM cells. Together, our results revealed that Cdc37 is downregulated in some relapsed MM patients, especially those that undergo BTZ-based therapy. Mechanistically, Cdc37 inhibition results in plasma cell immaturity and increased quiescent cell population, leading to BTZ resistance.

Cdc37 was previously reported as an overexpressed oncogene that mediates carcinogenesis by stabilizing the oncogenic kinases in a number of cancers. In this study, we also found that Cdc37 is upregulated in newly diagnosed MM patients compared with healthy individuals (data not shown). Notably, we also found that Cdc37 is downregulated in some relapsed MM patients, which seems paradoxical. To better understand the contradiction, we should underline the terms of clonal heterogeneity and clonal evolution of MM.

Due to the emergency of novel techniques, such as comparative genomic hybridization, genome sequencing, and multicolor in situ immunofluorescence hybridization, the concept of clonal heterogeneity and clonal evolution has gained great attention in the field of MM^38–40^. In some MM patients, several subclones already coexist and compete with each other within the tumor bulk at diagnosis. In the “Darwinian evolutionary model”, treatment can be considered as a selective pressure to eradicate the major subclone, which is chemosensitive. However, some small subclone, which is initially dormant and drug resistant, subsequently survives, expands, and gives rise to disease relapse. Thus, the source of disease recurrence is probably not the major subclone at diagnosis, but certain small drug-resistant subclones, whose genetic characteristics are easily “diluted” or even “buried” in bulk tumor. Therefore, the exploration of sequential samples, relapsed cases, or minimal residual disease is an important way to identify key genes associated with drug resistance. In our study, Cdc37 expression was explored in both sequential samples and relapsed cases. Overall, Cdc37 may be upregulated in the major subclone at diagnosis, which promotes the transformation to malignancy and accelerates the proliferation rate, thus facilitating the cytotoxicity of BTZ^33^. However, after the selective pressure of BTZ therapy, subclones with low expression of Cdc37, which are quiescent and drug insensitive, are selected out and become the dominant clones at relapse.

It is well established that Cdc37 depletion amplifies the antitumor effects of Hsp90 inhibitor^10,14^. However, in our study, Cdc37 suppression impairs the anti-MM activity of BTZ. This discrepancy is probably due to the different antitumor mechanisms of BTZ and Hsp90 inhibitor. The distinct characteristic of Cdc37 and Hsp90 in fostering client kinase activity underlies the ability of Cdc37 targeting to enhance the cytotoxicity of Hsp90 inhibitor. However, BTZ exerts its anti-MM effects in a very different way. It is believed that the antitumor activity of BTZ is due to the reversible inhibition of proteasome chymotrypsin-like activity. Proteasome inhibition prevents the clearance of large amounts of unfolded proteins, leading to endoplasmic reticulum overload, and triggering unfolded protein response, resulting in cell apoptosis eventually.

The differentiation status of MM is related to drug resistance, and the induction of differentiation has achieved great success in acute promyelocytic leukemia. Ig synthesis and secretion are prominent features of myeloma cells. With the maturation of plasma cells, the synthesis and secretion activities are gradually enhanced, thus raising the possibility that the differentiation degree of malignant plasma cells may have an impact on BTZ sensitivity. Previous studies suggested that BTZ-resistant MM cells contain greater proportions of less-differentiated cells, and BTZ resistance can be reversed by induced expression of plasma cell maturation markers^15,20,22^. In this study, we showed, for the first time, that Cdc37 expression correlates with Xbp1s, and that disrupting Cdc37 leads to reversal of plasma cell maturation through Xbp1s and consequently contributes to BTZ resistance in vitro and in vivo.

In summary, we find that Cdc37 downregulation may result in BTZ resistance through Xbp1s-mediated plasma cell immaturity in vitro and in vivo. Both CD38^–^CD138^–^pre-plasmablasts (Cdc37^−^Xbp1s^−^) and CD38^+^CD138^−^ plasmablasts (Cdc37^low^Xbp1s^low^) may evade BTZ treatment and subsequently survive, expand, and give rise to disease relapse. While the matured CD38^+^CD138^+^(Cdc37^high^Xbp1s^high^) plasma cells are sensitive to BTZ (Fig. 7f). Our findings reveal that Cdc37 may be a diagnostic biomarker for predicting BTZ sensitivity prior to treatment, and it is a significant formulation of clinical treatment plan.

## Supplementary information

Supplemental Figure
