# Supplementary material for: Cdc37 suppression induces plasma cell immaturation and bortezomib resistance in multiple myeloma via Xbp1s
Source: Oncogenesis. 2020 Mar 5;9(3):31. doi: 10.1038/s41389-020-0216-1 (PMC7058164; doi:10.1038/s41389-020-0216-1)
Supplement: Supplementary file 1 — Supplemental Figure [file 41389_2020_216_MOESM1_ESM.docx]

**
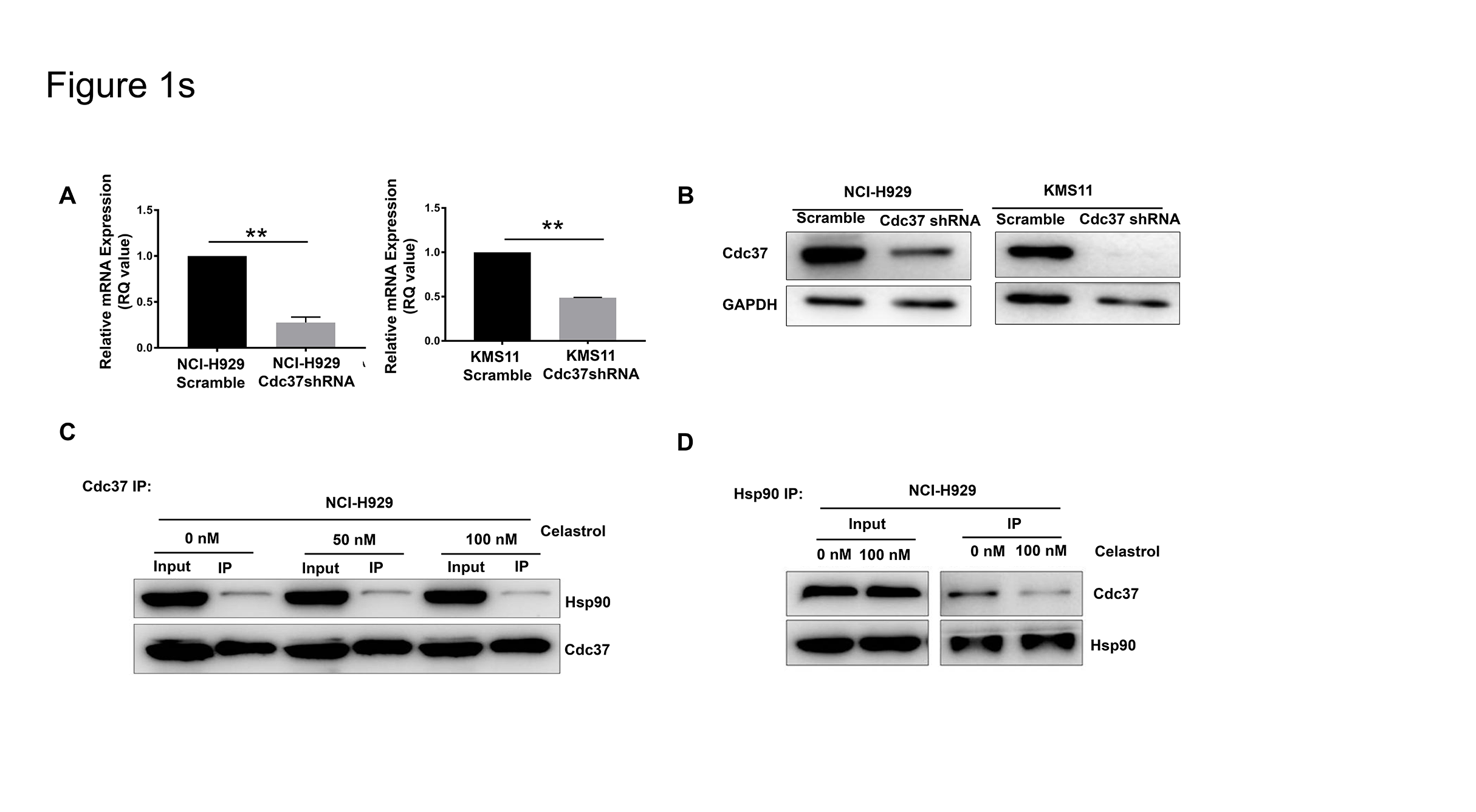
**

**Supplemental Figure 1. Cdc37 was suppressed by shRNA and Hsp90-Cdc37 interaction was disrupted by celastrol in MM cells**

(A) NCI-H929 and KMS11 cells were infected with scramble and Cdc37shRNA lentivirus. The Cdc37 gene expression was detected by qRT-PCR.

(B) NCI-H929 and KMS11 cells were infected with scramble and Cdc37shRNA lentivirus. The Cdc37 protein expression was detected by western blot.

(C) NCI-H929 cells were treated with indicated concentrations of celastrol for 48h and co-immunoprecipitation (IP) of Cdc37 protein was carried out. Co-IP using Cdc37 antibody shows that less Hsp90 were co-IP with Cdc37 in samples treated with 100nM celastrol.

(D) Co-IP using Hsp90 antibody shows that less Cdc37 was co-IP with Hsp90 in NCI-H929 cells treated with 100nM celastrol for 48h.

**
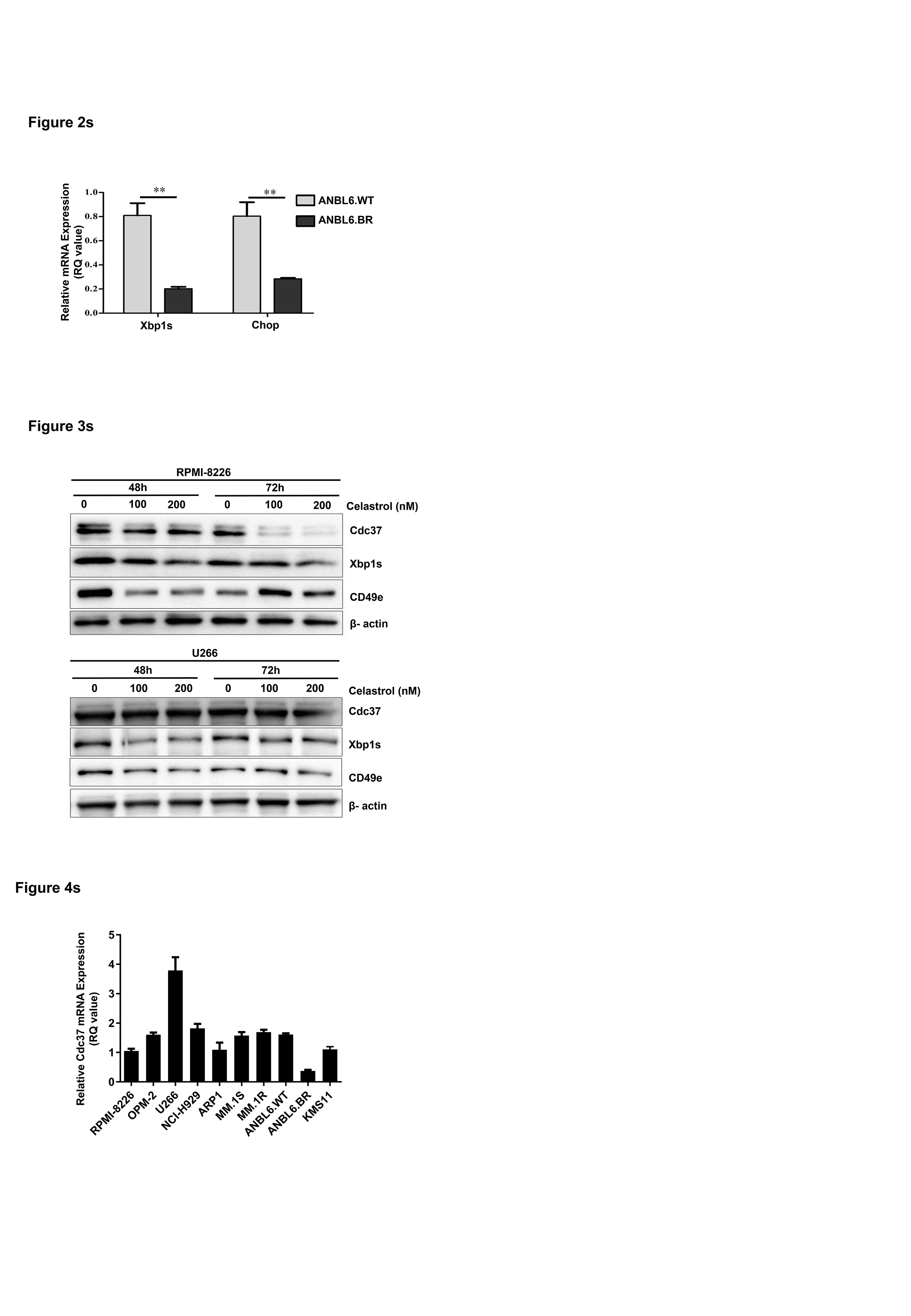
**

**Supplemental Figure 2. The critical transcription factors mediating plasma cell differentiation were down-regulated in BTZ resistant cell line**

Gene expression of Xbp1s and Chop was determined in ANBL6.WT and ANBL6.BR cells by qRT-PCR (** *p*<0.01)

**
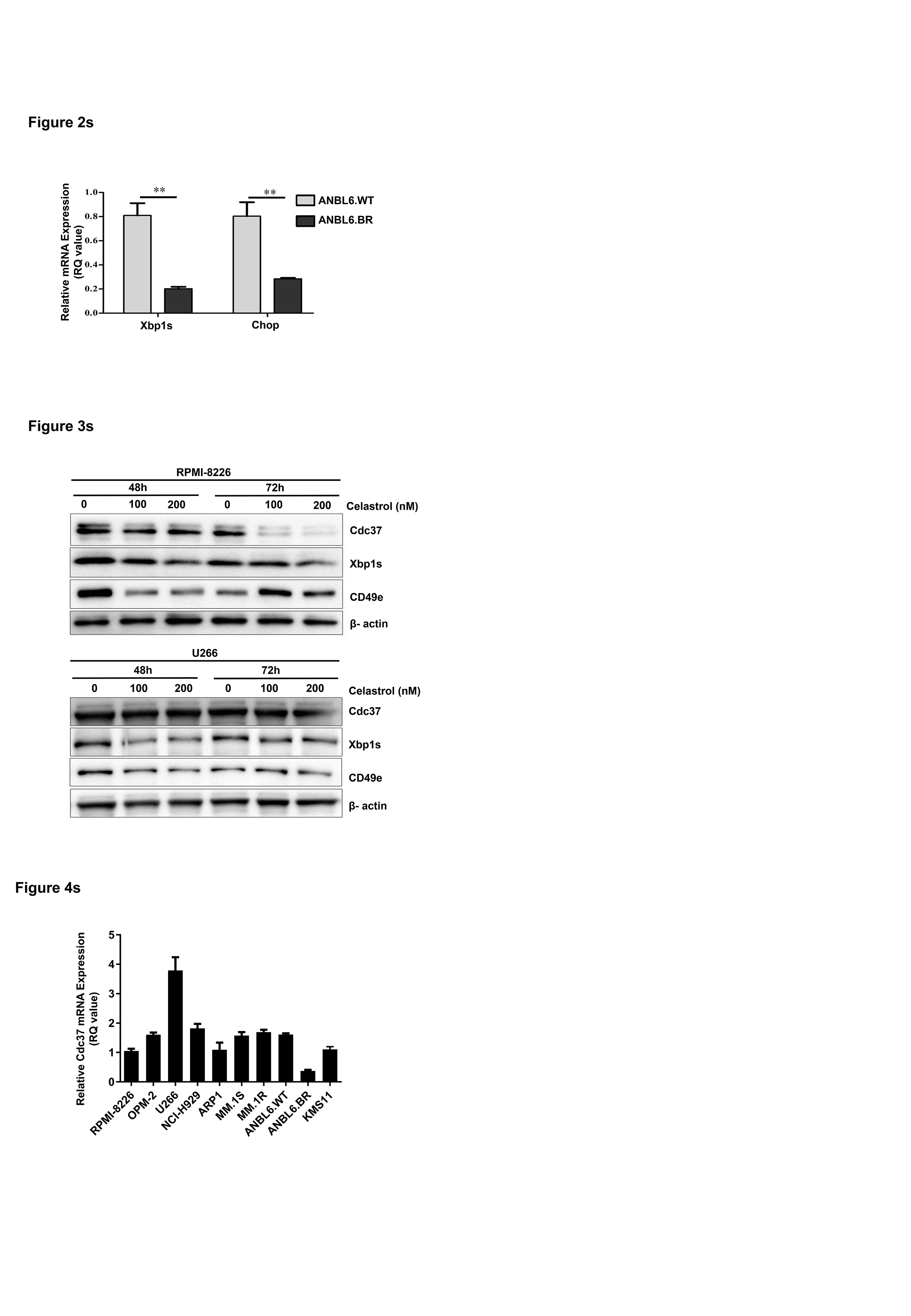
**

**Supplemental Figure 3. Interrupting Cdc37-Hsp90 interaction results in down-regulation of biomarkers of plasma cells maturation**

RPMI-8226 cells and U266 cells were treated with 100nM and 200nM celastrol for 48h and 72h, and the whole cell lysates were subjected to western blot for Cdc37 and Xbp1s.

**
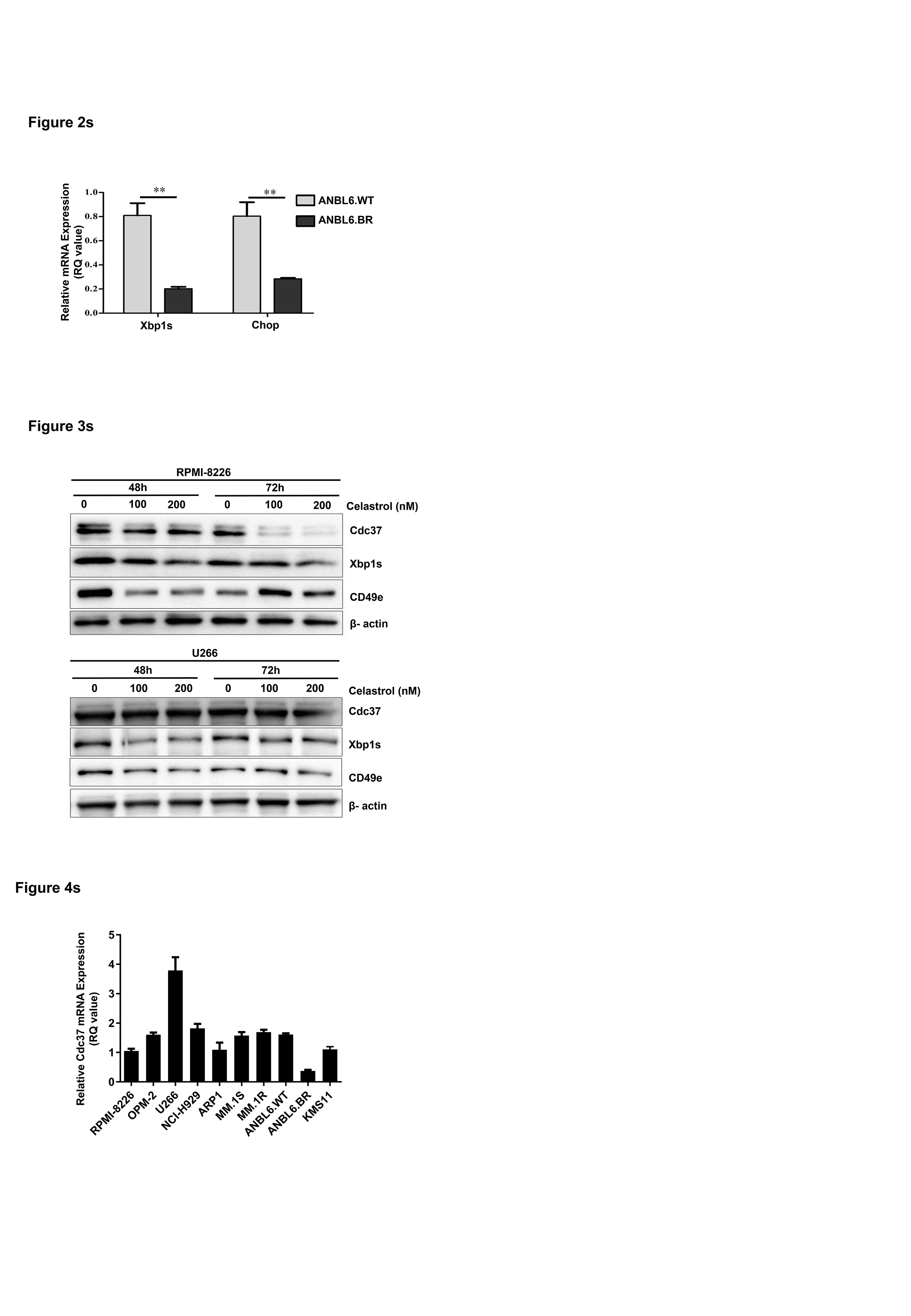
**

**Supplemental Figure 4. Cdc37 gene expression in MM cell lines**

Cdc37 gene expression was detected in MM cell line RPMI-8226, OPM-2, U266, NCI-H929, ARP1, MM.1S, MM.1R, ANBL6.WT, ANBL6.BR and KMS11 by qRT-PCR.
